# Structural network efficiency mediates the association between glymphatic function and cognition in mild VCI: a DTI-ALPS study

**DOI:** 10.3389/fnagi.2022.974114

**Published:** 2022-11-16

**Authors:** Hao Song, Zhao Ruan, Lei Gao, Dongwei Lv, Dong Sun, Zeng Li, Ran Zhang, Xiaoli Zhou, Haibo Xu, Junjian Zhang

**Affiliations:** ^1^Department of Neurology, Zhongnan Hospital of Wuhan University, Wuhan, China; ^2^Department of Radiology, Zhongnan Hospital of Wuhan University, Wuhan, China

**Keywords:** vascular cognitive impairment, glymphatic system, structural network, global efficiency, cerebral small vessel disease, post stroke cognitive impairment

## Abstract

**Background and objective:** Vascular cognitive impairment (VCI) can be caused by multiple types of cerebrovascular pathology and is considered a network disconnection disorder. The heterogeneity hinders research progress in VCI. Glymphatic failure has been considered as a key common pathway to dementia recently. The emergence of a new method, Diffusion Tensor Image Analysis Along the Perivascular Space (DTI-ALPS), makes it possible to investigate the changes of the glymphatic function in humans non-invasively. We aimed to investigate alterations of glymphatic function in VCI and its potential impact on network connectivity.

**Methods:** We recruited 79 patients with mild VCI, including 40 with cerebral small vessel disease cognitive impairment (SVCI) and 39 with post-stroke cognitive impairment (PSCI); and, 77 normal cognitive (NC) subjects were recruited. All subjects received neuropsychological assessments and multimodal magnetic resonance imaging scans. ALPS-index was calculated and structural networks were constructed by deterministic tractography, and then, the topological metrics of these structural connectivity were evaluated.

**Results:** The ALPS-index of VCI patients was significantly lower than that of NC subjects (*P* < 0.001). Multiple linear regression analysis showed that ALPS-index affects cognitive function independently (*β* = 0.411, *P* < 0.001). The results of correlation analysis showed that the ALPS-index was correlated with overall vascular risk factor burden (*r* = −0.263, *P* = 0.001) and multiple cerebrovascular pathologies (*P* < 0.05). In addition, global efficiency (Eg) of network was correlated with ALPS-index in both SVCI (*r* = 0.348, *P* = 0.028) and PSCI (*r* = 0.732, *P* < 0.001) patients. Finally, the results of mediation analysis showed that Eg partially mediated in the impact of glymphatic dysfunction on cognitive impairment (indirect effect = 7.46, 95% CI 4.08–11.48).

**Conclusion:** In both major subtypes of VCI, the ALPS-index was decreased, indicating impaired glymphatic function in VCI. Glymphatic dysfunction may affect cognitive function in VCI by disrupting network connectivity, and, may be a potential common pathological mechanism of VCI. ALPS-index is expected to become an emerging imaging marker for VCI.

## Introduction

Cerebrovascular disease is the second most common cause of dementia (Toledo et al., 2013). Vascular cognitive impairment (VCI) is a very comprehensive concept involving all forms and varying degrees of cognitive impairment associated with cerebrovascular disease, ranging from mild vascular cognitive impairment to vascular dementia (VaD; van der Flier et al., 2018). VCI involves various cerebrovascular pathological changes (Sachdev et al., 2014), and cerebral small vessel disease (CSVD) is the most common cause of VCI (Iadecola et al., 2019). In addition, stroke is also a very prevalent cause of VCI, 2–6 months after the acute cerebral ischemic event, 44% of patients were impaired in global cognition and 30%–35% of patients experienced impairment of individual cognitive domains (Lo et al., 2019). Due to the encompassing of a variety of cerebrovascular pathologies, the enormous heterogeneity of VCI has hindered research in this field, including the identification of valid biomarkers. Finding an underlying common mechanism leading to VCI is of great help in the diagnosis and treatment of VCI.

In recent years, the emergence of network neuroscience or graph theory has provided new insights into the neural basis of VCI. The brain is a complex connectome both functionally and structurally. Graph theoretical analysis allows to quantify the properties of brain networks and reveal important facets about the local and global organization of human brain architecture (Bullmore and Sporns, 2009). A growing body of evidence supports that VCI may be a disconnection disorder. This view considers that CSVD is a network-level rather than a focal disease (Ter Telgte et al., 2018), the association between CSVD neuroimaging lesions and cognitive deficits can be well explained by altered network organization (Tuladhar et al., 2016). In addition, network disconnection caused by stroke is also associated with post-stroke cognitive impairment (PSCI; Bournonville et al., 2018; Salvalaggio et al., 2020).

The glymphatic system is a functional waste clearance pathway in the brain discovered in recent years, which utilizes a unique perivascular channel system formed by astrocytes to clear soluble proteins and metabolic wastes from the central nervous system (Yu et al., 2019). In addition to waste-clearing function, the glymphatic system also helps distribute non-waste compounds such as glucose, lipids, and amino acids in the brain (Jessen et al., 2015). The astrocyte water channel and glymphatic system disorder will lead to the accumulation of metabolic waste, which has been shown to be related to aging (Kress et al., 2014) and various neurological diseases such as Alzheimer’s disease (Sweeney and Zlokovic, 2018), idiopathic normal pressure hydrocephalus (Bae et al., 2021), Parkinson’s disease (Ma et al., 2021) and so on. Cerebrovascular pathologies such as CSVD (Zhang W. et al., 2021) and stroke (Gaberel et al., 2014) have also been shown to be associated with glymphatic dysfunction. Recent insights suggest that the glymphatic system is a common final pathway in dementia and could potentially link the cardiovascular disease to neurodegenerative diseases (Nedergaard and Goldman, 2020). Therefore, it is a very attractive hypothesis that glymphatic dysfunction is a common underlying mechanism of cognitive impairment in various cerebrovascular pathologies, given that VCI is a network-level disorder, network disconnection may play an important role in this process.

Repeated contrast-enhanced magnetic resonance imaging based on intrathecal or intravenous gadolinium contrast agent has been routinely used to reflect glymphatic function in clinical studies, but this method is invasive and requires multiple scans (Taoka and Naganawa, 2020). As the movement of cerebrospinal and interstitial fluids forms the functional basis of the glymphatic system, diffusion tensor imaging may help reveal this process. In 2017, Taoka et al. (2017) proposed a new non-invasive method—“Diffusion Tensor Image Analysis Along the Perivascular Space” (DTI-ALPS) to research glymphatic function. Subsequent studies have shown a high reproducibility of the ALPS-index under specified MRI scan parameters (Taoka et al., 2022). This emerging noninvasive technique provides an attractive new approach to measure glymphatic function in the clinic and has been used in a growing number of studies. For example, a study using the DTI-ALPS approach have demonstrated impairment of the glymphatic function in multiple sclerosis and its association with more significant clinical disability (Carotenuto et al., 2022). In another study, the DTI-ALPS index could effectively reflect changes in glymphatic activity in patients with idiopathic normal pressure hydrocephalus (Bae et al., 2021).

Although the glymphatic system is considered to be the common final pathway in dementia and its relationship to neurodegenerative diseases such as AD has been demonstrated, few studies have focused on the glymphatic function in patients with VCI. To explore whether glymphatic dysfunction is an underlying common mechanism leading to VCI, and to identify a new imaging marker of VCI, this study used the non-invasive DTI-ALPS approach to investigate glymphatic function in VCI patients. Since VCI is a brain network disorder, we hypothesized that the impact of glymphatic impairment on cognitive function might be related to disruption of brain structural network connectivity. Therefore, using a graph-theoretic approach, we investigated the topological properties changes in the brain structural network of VCI patients and analyzed their association with glymphatic function. Finally, *via* mediation analysis, we explored the mediating role of the topological properties of brain structural networks in the association between glymphatic function and cognition in VCI.

## Subjects and Methods

### Participants

This study has been ethically approved (ClinicalTrials.gov ID: NCT04999813) and all participants gave written informed consent. In this study, 156 subjects were recruited from the Department of Neurology, Zhongnan Hospital of Wuhan University and the community. Considering that cerebral small vessel disease cognitive impairment (SVCI) and post-stroke cognitive impairment (PSCI) account for the vast majority of VCI patients, and the findings combining SVCI and PSCI are sufficiently representative, this study recruited 40 SVCI and 39 PSCI patients, for a total of 79 VCI patients. Normal cognition control (NC) group recruited 77 subjects, including 30 healthy controls (HC), 33 cerebral small vessel disease with normal cognition (SVDN), and 14 post-stroke cognitively normal (PSCN) controls. SVDN subjects had no history of stroke and met neuroimaging criteria for CSVD (Wardlaw et al., 2013); PSCN subjects had a history of stroke prior to 6 months. The diagnosis of VCI relies on neuropsychological assessment and neuroimaging. We diagnosed mild VCI in subjects with a Montreal Cognitive Assessment (MoCA) score <24, no impairment of independence of daily activities, and imaging findings meeting the VASCOG neuroimaging criteria (Sachdev et al., 2014). Among them, patients with cognitive impairment with a clear temporal relationship to stroke events and failure to fully recover within 6 months after stroke were classified as PSCI (Skrobot et al., 2018); patients meeting the following VASCOG imaging criteria were classified as SVCI: more than two lacunar infarcts outside the brainstem (1–2 lacunes may be sufficient if strategically placed or in combination with extensive white matter lesions) or extensive and confluent white matter lesions. All subjects were Chinese Han nationality, right-handed, aged 50–75 years old, and have more than 6 years of education.

This study excluded the following patients: (1) with a history of recent (within 6 months) or multiple strokes; (2) comorbid with other neurological diseases such as epilepsy, Parkinson’s disease and multiple sclerosis; (3) systemic diseases that may affect cognition, such as tumors and hypothyroidism; (4) head trauma or hemorrhage; (5) mental illness such as moderate to severe anxiety and depression; (6) drug or alcohol abuse; (7) visual or hearing impairment; and (8) unable to cooperate with MRI examination (Guo et al., 2022).

### Neuropsychological evaluations

All subjects underwent cognitive function assessment, including Mini-Mental State Examination (MMSE) and MoCA. MOCA is a test with a total score of 30 points, including multiple cognitive domains such as visuospatial, executive function, memory, attention, language, and orientation, and has good accuracy for the diagnosis and identification of VCI (Ghafar et al., 2019). The Hamilton Scale of Anxiety (HAMA) and Depression (HAMD) was used to assess the anxiety and depression of subjects by asking them a series of questions. Subjects with HAMA score >14 or HAMD >17 were excluded from the study. The Activities of Daily Living (ADL)/Instrumental Activities of Daily Living (IADL) scales, a tool for assessing independence in daily living, was used to differentiate the severity of VCI. Mild VCI was diagnosed in patients with VCI whose independence of daily living was unaffected or mildly affected (Skrobot et al., 2018). Framingham Stroke Risk Profile (FSRP; Bos et al., 2017) covers factors such as age, blood pressure, diabetes, atrial fibrillation, smoking, etc., and was used to measure the overall burden of vascular risk factors (VRFs).

### MRI protocols

The MR images were acquired using a 3.0 T MR scanner (Siemens Healthcare, Erlangen, Germany). T1 weighted images were acquired with a 3-D MP-RAGE sequence, the parameters were: TR = 6.7 ms, TE = 2.26 ms, flip angle = 9°, inversion time (TI) = 900 ms, field of view = 256 × 256 mm, voxel size = 1 × 1 × 1 mm, 176 sagittal slices. T2 FLAIR images were acquired with inversion recovery MATRIX sequence, the parameters were: TR = 6,000 ms, TE = 388 ms, echo train length = 848, bandwidth = 781 Hz/pixel, field of view = 256 × 256 mm, voxel size = 1 × 1 × 1 mm, 176 sagittal slices. Diffusion tensor images was acquired with an echo planar imaging sequence, the parameters were: TR = 9,200 ms, TE = 86 ms, field of view = 256 × 256 mm, voxel size = 2 × 2 × 2 mm, 72 axial slices, diffusion direction = 64, *b* = 0 and 1,000, phase encoding direction = posterior − anterior. Another b0 image with an opposite phase encoding direction was acquired to correct EPI distortions. The acquisition planes were adjusted so that the axial slices were aligned with the AC-PC line, and the brain was in a relatively standard position.

### ALPS-index

The principle that ALPS index could reflect glymphatic function is that, in the vicinity of the lateral ventricle body, the diffusion of water in the white matter mainly comes from the perivascular space of the medullary veins and the fiber tracts (projection fibers and association fibers) in white matter, and the direction of the medullary veins is perpendicular to the lateral ventricle wall and the direction of the two fiber tracts, thus allowing a near-independent analysis of the water diffusivity along the perivascular space (Taoka et al., 2017).

**Figure 1:**
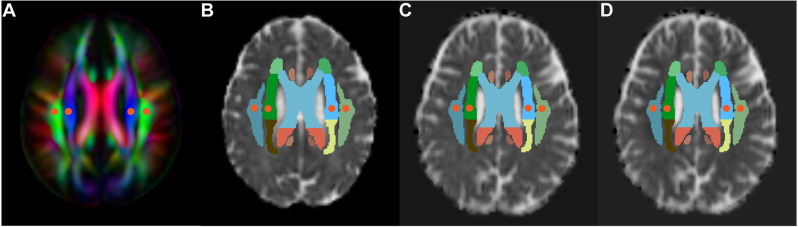
Demonstration of ROI placement. The placement of the ROIs (red circles with a diameter of 5 mm) was determined on the color-coded standard fractional anisotropy (FA) atlas, and these ROIs were placed in the bilateral projection fibers (blue area) and association fibers (green area) in FA atlas, respectively **(A)**. Then, these ROIs were placed on the diffusivity maps (spatially normalized) in the direction of the x-axis **(B)**, y-axis **(C)**, and z-axis **(D)**.

For the calculation of the ALPS-index, we refer to the improved mALPS calculation method by Zhang W. et al. (2021). Specifically, we obtained the diffusivity maps in the direction of the x-axis (Dx, right-left), y-axis (Dy, anterior-posterior) and z-axis (Dz, inferior-superior) of each subject using FSL software version 6.0^1^. Subsequently, the diffusivity maps in all directions were co-registered to the standard FA map template of the ICBM DTI-81 Atlas^2^. Since most patients have the same direction of medullary veins in the uppermost layer of the lateral ventricle body, the placement of ROI can be performed only based on DTI images without relying on SWI. Using a standard color-coded fractional anisotropy (FA) map, four spherical ROIs (5 mm in diameter) were placed on the projection fibers and association fibers in the uppermost layer of the lateral ventricle body, respectively (Figure 1). We checked the ROIs location for each subject and manually adjusted ROIs in some patients to ensure that all ROIs were accurately placed and avoided ischemic lesions. Then, the diffusivity in the ROIs in the three directions of x, y, and z-axis was extracted. The ALPS-index reflects the ratio of the water diffusivity along the space around the medullary vein to the diffusivity along other non-fiber running directions. The water diffusivity along the perivenous space includes the x-axis diffusivity within the projection fiber (Dx_projc) and association fiber (Dx_assoc), and the diffusivity in other non-fiber running directions includes the y-axis diffusivity within the projection fiber (Dy_proj) and the diffusivity of the z-axis within the association fiber (Dz_assoc). The calculation formula is:


$$\text{ALPS}-\text{index }=\frac{\text{mean}\left(\text{Dx}\_\text{proj},\text{ Dx}\_\text{assoc}\right)}{\text{ mean}\left(\text{Dy}\_\text{proj},\text{ Dz}\_\text{assoc}\right)\;}$$

The mean of the bilateral ALPS-index represented the overall glymphatic function of the subjects.

### Structural network construction and deterministic tractography

DWI data preprocessing was carried out using the PANDA toolbox (Cui et al., 2013) based on the MATLAB and FMRIB Software Library v5.0 following a default setting. The preprocessed and corrected data were subsequently used to estimate the structural tracts with deterministic tractography. The deterministic tractography was computed with main parameters including a FA value threshold of 0.2 and a turning angle threshold of 45° of the Fiber Assignment by Continuous Tracking (FACT) algorithm (Descoteaux et al., 2009).

Whole-brain structural connectivity was calculated with the deterministic tractography, using the AAL-90 parcellation in the MNI152 standard-space. Structural connectivity, including mean FA, fiber length and fiber number, between each pair of AAL brain regions were estimated and constructed into a structural connectivity matrix.

Further, complex brain network measures based on the structural connectivity matrix were calculated and contrasted between the groups with the GRETNA toolbox (Wang et al., 2015). Considering a potential dysconnectivity nature of the VCI, we therefore only calculated the small-worldness [sigma and global shortest path length (Lp)], network efficiency [global efficiency (Eg), and local efficiency (Eloc)] measures, and clustering coefficient (Cp).

### Assessment of CSVD features

Neuroimaging features of CSVD include recent small subcortical infarct, white matter hyperintensity (WMH), lacune, enlarged perivascular space (EPVS), cerebral microbleed and brain atrophy (Wardlaw et al., 2013). Lacune, EPVS, and WMH were manually marked independently by two experienced radiologists (ZR and LG) without clinical information. Labeling of lacune was performed on 3D-T1. A lacune is defined as a subcortical round or oval fluid-filled cavity with a signal similar to that of CSF, ranging in diameter from 3 to 15 mm. The number of lacunes was recorded. Evaluation of EPVS and WMH was performed on the T2-Flair imaging. WMH is defined as white matter with hyperintensity on T2-Flair images and equal or hypointense on 3D-T1 images, we calculated WMH volume and performed total intracranial volume (TIV) normalization. EPVS is defined as a small, clear structure of the CSF signal, that is hypointense on T2-Flair without a surrounding hyperintensity ring, and these structures are aligned with the orientation of the perforating vessels. The number of EPVS was recorded and scored (Raposo et al., 2019). The EPVS in the basal ganglia and the semiovale center were scored as follows: 0 = no EPVS, 1 = 1–10 EPVS, 2 = 11–20 EPVS, 3 = 21–40 EPVS, 4 = >40 EPVS. Microbleed and brain atrophy were not evaluated in this study.

### Statistical analysis

All statistical analyses involved in this study were performed in SPSS software version 25 (IBM, United States). Depending on the data distribution, differences between the two groups were compared using t-test, *χ*^2^ test, or Wilcoxon Man-Whitney nonparametric test. The correlation analysis between variables uses Pearson’s correlation analysis or Spearman’s correlation analysis. Univariate and multivariate binary logistic regression was used to analyze the relationship between ALPS-index and VCI, controlling for confounders. Variables with *P* < 0.1 in univariate logistic regression or considered clinically relevant for VCI were included as covariates, along with the ALPS-index in percentile, into the multivariate logistic regression model. The correlation of multiple variables with cognition was analyzed using multiple linear regression, only variables with *P* < 0.01 were included in the regression model, and variance inflation factors (VIF) were calculated to assess the degree of multicollinearity. In the multiple regression model, each variable has a VIF <3, so there is no multicollinearity. Mediation analysis was performed using PROCESS macro 4.1 (Hayes, 2009) to further explore the relationship between ALPS-index, brain network topological measures, and cognition. When the indirect effect’s 95% confidence interval (95% CI) does not include 0, it means that there is a significant mediation effect, controlling for age, gender, and education as confounding factors. *P* < 0.05 was considered to be statistically significant.

## Results

### Demographics and clinical characteristics

A total of 156 subjects were included in this study, with an average age of 61.07 ± 4.97, of whom 45 (28.8%) were female. Table 1 presents demographic information for all subjects (divided into VCI and NC). Among all subjects, 88 (56.4%) had hypertension, 57 (36.5%) had diabetes, 78 (50%) had hyperlipidemia, and 72 had a history of smoking (46.1%). In addition, the demographics and clinical characteristics of SVDN and SVCI, PSCN, and PSCI are presented in Supplementary Tables 1, 2.

**Table 1:** Demographics and clinical characteristics.

**Characteristics**	**NC (*N* = 77)**	**VCI (*N* = 79)**	**t/χ^2^/Z**	***P*-value**
Age, y, mean ± SD	60.48 ± 4.51	61.65 ± 5.35	-1.47	0.144
Female, *n* (%)	25 (32.4%)	20 (25.3%)	0.97	0.324
Education, y, mean ± SD	12.84 ± 2.52	12.47 ± 2.71	0.90	0.372
Hypertension, *n* (%)	32 (41.5%)	56 (70.8%)	13.64	<0.001*
Hyperlipemia, *n* (%)	46 (59.7%)	32 (40.5%)	5.77	0.016*
Diabetes, *n* (%)	22 (28.5%)	35 (44.3%)	4.16	0.041*
History of smoking, *n* (%)	25 (32.4%)	47 (59.4%)	11.46	<0.001*
FSRP score, mean ± SD	6.69 ± 3.65	10.46 ± 4.30	-5.90	<0.001*
MoCA, mean ± SD	26.38 ± 1.60	16.10 ± 4.93	17.42	<0.001*
Lacune, *n*, median (interquartile range)	0 (0–1)	4 (2–8)	-7.98	<0.001*
WMH volume, ml, median	0.45 (0.17–1.58)	4.25 (1.45–11.99)	-6.99	<0.001*
Basal ganglia EPVS score, median (interquartile range)	2 (1–2)	2 (2–2)	-2.93	0.003*
Centrum semiovale EPVS score, median (interquartile range)	1 (0–1)	1 (0–1)	-1.108	0.268

NC, normal cognitive control; VCI, vascular cognitive impairment; FSRP, Framingham Stroke Risk Profile; MoCA, Montreal Cognitive Assessment; WMH, white matter hyperintensities; EPVS, enlarged perivascular spaces; SD, standard deviation; *differences are statistically significant.

### Association of ALPS-index with VCI

**Figure 2:**
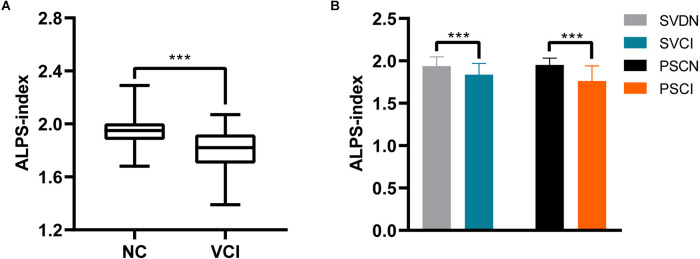
Differences of ALPS-index between NC and VCI groups **(A)**. Differences of ALPS-index between SVDN and SVCI, PSCN and PSCI **(B)**. NC, normal cognitive control; VCI, vascular cognitive impairment; SVDN, cerebral small vessel disease with normal cognition; SVCI, cerebral small vessel disease cognitive impairment; PSCN, post-stroke cognitively normal; PSCI, post-stroke cognitive impairment; ALPS, analysis along the perivascular space. ***P < 0.001.

First, the mean ALPS-index of VCI patients was 1.80 ± 0.16, which was significantly lower than the ALPS-index of NC subjects of 1.95 ± 0.11 (*P* < 0.001; Figure 2A). Then, analysis of the two subtypes of VCI showed that SVCI patients had a lower ALPS-index relative to SVDN subjects (1.84 vs. 1.94, *P* = 0.001), and the ALPS-index of PSCI patients was significantly lower than that of PSCN subjects (1.76 vs 1.95, *P* < 0.001; Figure 2B).

We then performed multivariate binary logistic regression analyses to eliminate the influence of confounding factors on the results. For better clinical interpretation, we used the percentage form of ALPS-index when performing logistic regression. The crude OR of ALPS-index (%) for VCI was 0.914 (95% CI 0.884–0.945, *P* < 0.001). After adjusting for potential confounders, the results of logistic regression showed that ALPS-index was an independent protective factor for VCI, and the reduction of ALPS-index (%) was associated with the risk of VCI (model 2: OR 0.912, 95% CI 0.881–0.944, *P* < 0.001; model 3: OR 0.868, 95% CI 0.817–0.921, *P* < 0.001; Table 2). Similar results were also found in the two main subtypes of VCI, SVCI, and PSCI (Table 2).

**Table 2:** Univariate and multivariate logistic analyses of the association between ALPS-index and VCI, SVCI, and PSCI.

**ALPS-index (%)**	**Model 1, unadjusted**			**Model 2^a^**			**Model 3^b^**	
	**OR (95% CI)**	***P***		**OR (95% CI)**	***P***		**OR (95% CI)**	***P***
VCI	0.914 (0.884–0.945)	<0.001		0.912 (0.881–0.944)	<0.001		0.868 (0.817–0.921)	<0.001
SVCI	0.937 (0.893–0.976)	0.003		0.927 (0.885–0.972)	0.002		0.863 (0.781–0.954)	0.004
PSCI	0.900 (0.838–0.966)	0.002		0.890 (0.825–0.960)	0.002		0.816 (0.712–0.935)	0.003

ALPS, analysis along the perivascular space; VCI, vascular cognitive impairment; SVCI, cerebral small vessel disease cognitive impairment; PSCI, post-stroke cognitive impairment; OR, odds ratio; CI, confidence interval. ^a^Adjusted for age, gender, and education. ^b^Adjusted for age, gender, education, hypertension, hyperlipemia, diabetes, history of smoking, and Framingham Stroke Risk Profile.

### Association of ALPS-index with cognition

Correlation analysis showed that ALPS-index was associated with cognitive function in both SVCI patients (*r* = 0.33, *P* = 0.037) and PSCI patients (*r* = 0.50, *P* = 0.001).

We included variables in demographics, VRFs, and CSVD features correlated with MoCA score (*P* < 0.01), together with ALPS-index, into the multiple linear regression model. After controlling for the influence of other variables, ALPS-index was correlated with the MoCA score (β = 0.411, *P* < 0.001; Table 3), indicating that ALPS-index was an independent influencing factor of cognitive function.

**Table 3:** Multivariate analyses of MoCA.

**Variables**	**β**	***P*-value**
Age	0.002	0.974
FSRP score	−0.327	<0.001
Hypertension	0.048	0.507
Diabetes	0.003	0.966
History of smoking	0.069	0.299
ALPS-index	0.411	<0.001
Lacune	−0.345	<0.001
WMH	0.103	0.189

MoCA, montreal cognitive assessment; FSRP, Framingham Stroke Risk Profile; ALPS, analysis along the perivascular space; WMH, white matter hyperintensities; β, standardized coefficient.

### Relationship between ALPS-index and VRFs and CSVD imaging features

Since the alteration of ALPS-index in VCI patients and its correlation with cognition have been confirmed, we further explored the association of VRFs or cerebrovascular pathological changes with ALPS-index. The results showed that the FSRP score, which reflects the overall burden of VRFs, was correlated with the ALPS-index (*r* = −0.263, *P* = 0.001). Analysis of specific demographic factor and VRF revealed that age (*r* = −0.352, *P* < 0.001), hypertension (*r* = −0.559, *P* < 0.001), and diabetes (*r* = −0.476, *P* < 0.001) correlated with the ALPS-index. Gender (*r* = 0.048, *P* = 0.554), hyperlipidemia (*r* = 0.095, *P* = 0.240), and smoking history (*r* = −0.138, *P* = 0.086) were not associated with ALPS-index (Table 4).

**Table 4:** Relationship between ALPS-index and VRFs and CSVD imaging features.

**Variables**	**Pearson r or Spearman r**	***P*-value**
Age	−0.353	<0.001
Gender	0.048	0.554
FSRP score	−0.263	0.001
Hypertension	−0.559	<0.001
Hyperlipemia	0.095	0.240
Diabetes	−0.476	<0.001
History of smoking	−0.138	0.086
Lacune	−0.447	<0.001
WMH volume	−0.464	<0.001
Basal ganglia EPVS score	−0.220	0.006
Centrum semiovale EPVS score	−0.190	0.018

ALPS, analysis along the perivascular space; FSRP, Framingham Stroke Risk Profile; WMH, white matter hyperintensities; EPVS, enlarged perivascular spaces.

With regard to imaging features of CSVD, normalized WMH volume (*r* = −0.464, *P* < 0.001), number of lacune (*r* = −0.447, *P* < 0.001), basal ganglia EPVS score (*r* = −0.220, *P* = 0.006), and semiovale center EPVS score (*r* = −0.190, *P* = 0.018) were all related to the ALPS-index (Table 4).

### The relationship between structural network connectivity and ALPS-index

**Figure 3:**
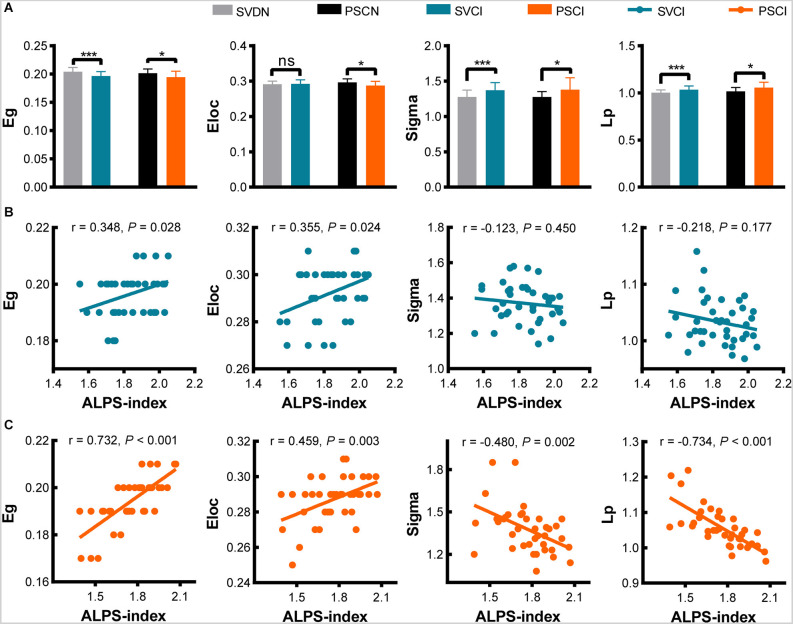
Differences of network topology metrics (Eg, Eloc, Sigma, Lp) between SVDN and SVCI, PSCN, and PSCI **(A)**. Correlation between ALPS-index and network topology metrics in SVCI **(B)** and PSCI **(C)**. *P < 0.05, ***P < 0.001; ns, no statistical difference; Eg, global efficiency; Eloc, local efficiency; Lp, global shortest path length; ALPS, analysis along the perivascular space; SVDN, cerebral small vessel disease with normal cognition; SVCI, cerebral small vessel disease cognitive impairment; PSCN, post-stroke cognitively normal; PSCI, post-stroke cognitive impairment.

Our results show that brain structural connectivity is markedly impaired in VCI patients. Compared with SVDN subjects, SVCI patients had lower Eg (0.197 vs. 0.204, *P* < 0.001), higher Sigma (1.37 vs. 1.27, *P* < 0.001), and Lp (1.03 vs. 1.00, *P* = 0.001), while Eloc has no statistical difference (0.292 vs. 0.291, *P* = 0.587); compared with PSCN subjects, PSCI patients had lower Eg (0.195 vs. 0.201, *P* = 0.031), lower Eloc (0.29 vs. 0.30, *P* = 0.017), and higher Sigma (1.38 vs. 1.28, *P* = 0.032) and Lp (1.06 vs. 1.02, *P* = 0.022; Figure 3A). There were no differences in Cp between the SVCI and SVDN (0.199 vs. 0.195, *P* = 0.071), as well as, PSCI and PSCN groups (0.197 vs. 0.199, *P* = 0.717, not shown in the figure).

Subsequently, we investigate the correlation of ALPS-index with network topology measures. In SVCI patients, ALPS-index was associated with Eg (*r* = 0.348, *P* = 0.028) and Eloc (*r* = 0.355, *P* = 0.024; Figure 3B); in PSCI patients, ALPS-index was correlated with Eg (*r* = 0.732, *P* < 0.001), Eloc (*r* = 0.459, *P* = 0.003), Sigma (*r* = −0.480, *P* = 0.002), and Lp (*r* = −0.734, *P* < 0.001; Figure 3C). It can be found that network efficiency shows a correlation with ALPS-index in both VCI subtypes.

### The associations between ALPS-index, Eg, and cognition

**Figure 4:**
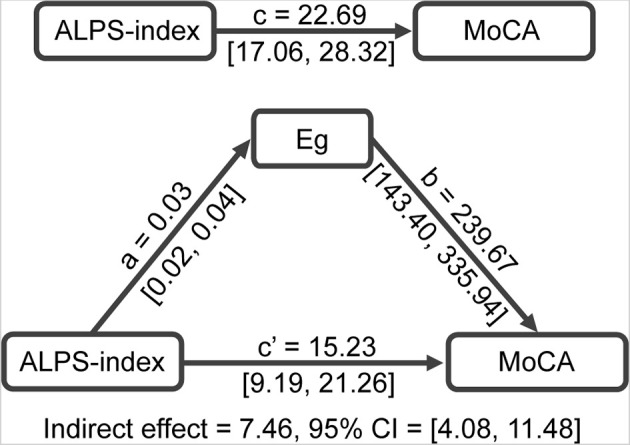
Mediation analysis between ALPS-index (X) and MoCA score (Y), with Eg as mediator (M). ALPS, analysis along the perivascular space; Eg, global efficiency; MoCA, Montreal Cognitive Assessment.

To further explore the relationship between ALPS-index, network topological measures, and cognition, we performed a mediation analysis on them, with ALPS-index as the independent variable (X) and MoCA score as the dependent variable (Y). Since in both SVCI and PSCI patients, Eg was significantly decreased and was related to ALPS-index, we selected Eg as a mediating variable (M). The results of mediation analysis showed that Eg mediated the relationship between ALPS-index and MoCA score (indirect effect = 7.46, 95% CI 4.08–11.48; Figure 4), with a mediating effect ratio of 33%.

## Discussion

In this study, we reveal for the first time the relationship between glymphatic function reflected by the DTI-ALPS index, the properties of brain structure network, and cognitive function in VCI patients. The main findings of this study are as follows: (1) the ALPS-index of VCI patients was significantly reduced, which means there is an impairment of glymphatic function in VCI; (2) vascular risk factor burden and markers of CSVD, including lacune, WMH, and EPVS, were associated with ALPS-index; and (3) ALPS-index is related to cognitive function and brain structural network connectivity disruption in VCI patients, and the global topological metrics (Eg) of the structural network mediated the relationships between glymphatic function and cognition in VCI patients.

Cerebrospinal fluid (CSF) and interstitial fluid (ISF) are constantly exchanged in our brains. This exchange is facilitated by the convective inflow of CSF along the periarterial space. A combination of arterial pulsation, respiration, and CSF pressure gradients drives CSF from the subarachnoid space into the penetrating arterial perivascular space. The cerebral vasculature is surrounded by astrocyte endfeet, and aquaporin protein-4 (AQP4) expressed at the end of the endfeet drives the transport of CSF into the brain parenchyma. After entering the parenchyma, the CSF pushes the ISF and solutes toward the perivenous space for eventual draining from the CNS *via* the meningeal lymphatic vessels. This process is accompanied by rapid exchange of fluids and solutes between CSF and ISF, and we refer to this polarized macroscopic system of convective fluid fluxes as the glymphatic system (Jessen et al., 2015; Lv et al., 2021). Under physiological conditions, the convective influx of CSF in the glymphatic pathway is balanced by the peripheral outflow of ISF, thereby eliminating toxic metabolites, including Aβ, in neuronal cells. Obstruction of the glymphatic clearance pathway will lead to the deposition of metabolic wastes and lead to a series of pathophysiological changes, and are associated with various central nervous system diseases (Rasmussen et al., 2018). In the past, studies of human brain glymphatic clearance have used repetitive contrast-enhanced magnetic resonance imaging based on intrathecal gadolinium injections, which has limited application due to its invasive nature. Since the new non-invasive glymphatic function detection technology DTI-ALPS was proposed, it has been adopted by quite a few studies. Recent studies have confirmed that the ALPS-index is closely related to glymphatic clearance detected using classical methods, with high inter- and intra-observer correlation coefficients (Zhang W. et al., 2021), this suggests that the ALPS-index can be used as a robust and reliable indicator for measuring glymphatic function.

In this study, by using the DTI-ALPS approach, we found that glymphatic function impairment was present in both SVCI and PSCI, the two major subtypes of VCI. As the most common subtype of VCI, CSVD is usually secondary to aging or other vascular risk factors, and studies have shown that CSVD is associated with dysfunction of the glymphatic system (Mortensen et al., 2019) and may exacerbate the progression of neurodegeneration (Nedergaard and Goldman, 2020). In a rat MMI model mimicking the VCI state by multiple microinfarcts, multiple microinfarcts (MMIs) lead to delayed infiltration of CSF into the brain parenchyma through perivascular pathways and delay in waste clearance, resulting in glymphatic system dysfunction, which may play an important role in MMI-induced axonal damage and cognitive deficits (Venkat et al., 2017). In addition, chronic cerebral hypoperfusion is also associated with significant white matter damage, cognitive impairment, and impaired glymphatic function (Cao et al., 2022). Furthermore, we found in our study that in the context of prior ischemic stroke, patients with cognitive deficits had significant differences in glymphatic function compared with those with normal cognition; this suggests an essential role of glymphatic function in the occurrence of PSCI. In animal studies, it is widely accepted that ISF clearance is reduced after ischemic stroke (Lv et al., 2021). After ischemic stroke, CSF circulation in the glymphatic system is impaired, and clearance of amyloid deposits in the infarct core and perivascular space is delayed (Arbel-Ornath et al., 2013). However, studies have shown that post-stroke damage to the glymphatic system is transient and recovers gradually over time (Toh and Siow, 2021). During this process, a good function of the glymphatic system is beneficial to the long-term prognosis of ischemic stroke, and some scholars consider that the glymphatic function is related to post-stroke dementia (PSD; Pantoni, 2017). Some researchers believe that the deposition and hyperphosphorylation of tau are the main pathophysiological mechanisms of PSD, studies have shown that chronic cerebral hypoperfusion may aggravate cognitive impairment in rats with acute ischemic stroke by disrupting the clearance of Aβ and tau in the glymphatic system (Back et al., 2020), this indicates that glymphatic dysfunction is a risk factor for the development of PSD. These results suggest that dysfunction of glymphatic clearance may be a potential common mechanism of cognitive impairment caused by multiple cerebrovascular pathologies, strengthening the link between vascular pathogenic factors and neurodegeneration.

Patients with VCI are often accompanied by a higher burden of vascular risk factors, and several vascular risk factors, including hypertension and diabetes, are closely related to VCI. It is generally believed that long-term VRFs burden will cause a variety of cerebrovascular pathologies, including atherosclerosis, small vessel wall degeneration, etc., and then cause a series of downstream pathophysiological changes, thereby affecting cognition. In this regard, studies of the glymphatic system provide new insights. Alteration of glymphatic function has been observed in animal models of VRFs. Hypertension in particular causes hypertrophy of vascular smooth muscle cells, stiffening the arterial wall and inhibiting its pulsatility and compliance, thereby reducing perivascular fluid flow (Mestre et al., 2017). In a rat model of spontaneous hypertension, it was found that the perivascular space was significantly enlarged and the inflow and outflow function of the glymphatic system was significantly reduced (Xue et al., 2020). Similarly, diabetes has been shown to disturb the glymphatic system (Zhang Y. et al., 2019). Consistent with previous studies (Zhang et al., 2021), our results suggest that hypertension and diabetes are associated with the ALPS-index. Furthermore, we also found that the overall burden of VRFs as reflected by FSRP was highly correlated with the ALPS-index. As we have seen, a heavier burden of VRFs leads to glymphatic impairment and affects cognition, suggesting that glymphatic dysfunction may play a role in the contribution of VRFs to cognitive impairment.

Now that there is a definite reduction in the ALPS-index in patients with SVCI, we sought to figure out which specific CSVD neuroimaging features were associated with ALPS-index. Therefore, in this study, WMH, lacune, and EPVS were evaluated, and our results showed that no matter the lacune, WMH or EPVS were related to the ALPS-index. The perivascular space, a structure known to play an important role in glymphatic transport, can be seen as a low-resistance highway for CSF influx (Jessen et al., 2015), and current views interpret fluid stasis in the perivascular space as a sign of glymphatic dysfunction. EPVS is associated with abnormal collagen deposition in the downstream venous system, and severe EPVS pathology is a marker of cognitive decline and increased risk of dementia (Paradise et al., 2021). In terms of WMH, dysfunction of certain components of the glymphatic system directly impacts oligodendrocytes, which in turn lead to the demyelinating changes that manifest as WMH on MRI (Marignier et al., 2010). In turn, CSVD can also lead to glymphatic dysfunction. Animal studies have found that small, discrete ischemic lesions undermine glymphatic function throughout the brain, and the capture of solutes in these lesions may promote protein aggregation and neuroinflammation, ultimately leading to white matter damage and neurodegeneration (Wang et al., 2017). However, the causal relationship between CSVD and glymphatic dysfunction is still unclear. Based on the available evidence, CSVD and glymphatic dysfunction may promote and cause each other.

The view that VCI is a network disruption-related disease has been widely recognized. Our results also corroborate this view, in both VCI subtypes, Eg was significantly different from NC subjects, and PSCI patients were also accompanied by elevated small-world measures (Sigma and Lp), suggesting more extensive network damage in PSCI. Eg is an indicator to assess the efficiency of network information transfer, and previous studies have shown that the correlation between the CSVD and cognitive impairment is mediated by Eg decline (Boot et al., 2020). Eg was significantly associated with psychomotor speed and trends in cognitive index changes in CSVD patients and played an important role in cognitive decline in CSVD (van Leijsen et al., 2019). In addition, impairment of structural network connectivity predicts behavioral and cognitive deficits after stroke (Salvalaggio et al., 2020). The perspective of brain network connectivity allows VCI to break free from the shackles of complex pathological heterogeneity and provides a mechanism explanation closer to the essence of cognitive science. Our study found that ALPS-index is related to topology metrics such as network efficiency, which seems very plausible. The communication of information in the brain network depends on the white matter fiber bundles as the structural foundation, and the damage to the white matter microstructure will lead to the disruption of the network. Oligodendrocytes, which make up the myelin sheath of the central nervous system (CNS) and wrap neuronal axons, play an active role in the maintenance and repair of white matter (Dewar et al., 2003). Oligodendrocytes are particularly vulnerable to the accumulation of metabolic waste, so glymphatic clearance dysfunction directly impacts oligodendrocytes and leads to demyelination (Carotenuto et al., 2022), which is bound to affect information transmission in the brain network. To further explore the relationship between glymphatic dysfunction, network connectivity, and cognition, we performed a mediation analysis, which showed that Eg partially mediated the impact of glymphatic function on cognitive impairment. This confirms that glymphatic dysfunction contributes to cognitive impairment in VCI patients, at least in part, through reduced efficiency of brain network information transfer, revealing the underlying mechanism of cognitive impairment caused by glymphatic dysfunction from the level of human research. Given that ALPS-index is closely related to VRFs burden and cerebrovascular pathologies, we speculate that VRFs burden and cerebrovascular pathologies may affect network connectivity by disrupting glymphatic clearance, thereby causing cognitive impairment. Thus, glymphatic dysfunction provides a new hub in the association between various vascular pathogenic factors and cognitive impairment.

There are several limitations to this research. First, due to the small sample size, this study did not include other VCI patients except SVD and PSCI. Although SVCI and PSCI account for the majority of VCI, the glymphatic function of VCI patients with other pathological types still needs further verification. Second, we included a small number of PSCN subjects, which may have affected the statistical analysis results. Long-term changes in glymphatic function remain unclear for subjects with a history of stroke, although we included all subjects 6 months after the stroke event. Finally, for the assessment of cognition, we only used the MoCA scale without a detailed assessment of multiple cognitive domains, which is not conducive to reflect the subjects’ comprehensive cognitive function.

## Conclusion

By using the DTI-ALPS method, this study found significant glymphatic dysfunction in VCI and its major subtypes, which may affect cognitive function through disruption of brain structural networks connectivity. These findings support glymphatic dysfunction as an underlying common pathophysiological mechanism in the development of VCI, and the ALPS-index is expected to be an emerging VCI neuroimaging marker, which needs further validation in follow-up studies.

## Data Availability Statement

The raw data supporting the conclusions of this article will be made available by the authors, without undue reservation.

## Ethics Statement

The studies involving human participants were reviewed and approved by Medical Ethics Committee, Zhongnan Hospital of Wuhan University. Board Affiliation: Zhongnan Hospital of Wuhan University. The patients/participants provided their written informed consent to participate in this study.

## Author Contributions

HS contributed to study conception, design, subject recruitment, data analysis, and manuscript drafting. ZR was responsible for image acquisition and preprocessing, CSVD features labeling, and data analysis. LG contributed to the calculation of ALPS-index and network topology metrics. DL, DS, ZL, and RZ involved in subject recruitment and cognitive assessment. XZ involved in image acquisition. HX involved in providing the necessary instruments for the study and technical support. JZ contributed to study conception, design, revising the manuscript, and funding acquisition. All authors contributed to the article and approved the submitted version.

## Funding

This study was supported by The Central Government Guiding Funds for Local Science and Technology Development in Hubei Province of China (grant number 2020ZYYD014, JZ).

## Footnotes

^1^ https://fsl.fmrib.ox.ac.uk/fsl/fslwiki/

^2^ https://www.loni.usc.edu/research/atlases
