# Supplementary material for: Structural network efficiency mediates the association between glymphatic function and cognition in mild VCI: a DTI-ALPS study
Source: Front Aging Neurosci. 2022 Nov 16;14:974114. doi: 10.3389/fnagi.2022.974114 (PMC9708722; doi:10.3389/fnagi.2022.974114)
Supplement: Supplementary file 1 [file Table_1.pdf]

Supplementary Material

1 Supplementary Tables

Supplementary table 1 Demographics and Clinical Characteristics of SVDN and SVCI

**Characteristics**	**SVDN** (N = 33)	**SVCI** (N = 40)	**t / χ^2 / Z**	***P*-value**
Age, y, mean ± SD	61.33 ± 4.47	61.03 ± 5.23	0.27	0.790
Female, n (%)	9 (27.2%)	11 (27.5%)	0.00	1.000
Education, y, mean ± SD	12.64 ± 2.70	12.15 ± 2.53	0.79	0.430
Hypertension, n (%)	15 (45.4%)	29 (72.5%)	5.52	0.019*
Hyperlipemia, n (%)	22 (66.6%)	16 (40%)	5.15	0.023*
Diabetes, n (%)	10 (30.3%)	17 (42.5%)	1.15	0.283
History of smoking, n (%)	14 (42.4%)	21 (52.5%)	0.74	0.391
FSRP score, mean ± SD	7.55 ± 3.73	9.73 ± 3.73	-2.48	0.015*
MoCA, mean ± SD	26.30 ± 1.65	17.35 ± 4.40	11.07	<0.001*
Lacune, n, median (interquartile range)	0 (1-1)	2 (1-6.75)	-4.75	<0.001*
WMH volume, mL, median	0.77 (0.27-1.62)	2.39 (0.78-10.31)	-3.44	0.001*
Basal ganglia EPVS score, median (interquartile range)	2 (1-2)	2 (1-2)	-1.55	0.120
Centrum semiovale EPVS score, median (interquartile range)	1 (0-1)	1 (0-1)	-0.87	0.385

SVDN, cerebral small vessel disease with normal cognition; SVCI, cerebral small vessel disease cognitive impairment; FSRP, Framingham Stroke Risk Profile; MoCA, Montreal Cognitive Assessment; WMH, white matter hyperintensities; EPVS, enlarged perivascular spaces; SD, standard deviation; *, the difference is statistically significant.

Supplementary table 2 Demographics and Clinical Characteristics of PSCN and PSCI

**Characteristics**	**PSCN**<br>**(N = 14)**	**PSCI**<br>**(N = 39)**	**t / χ^2 / Z**	***P*-value**
Age, y, mean ± SD	61.50 ± 5.46	62.28 ± 5.46	-0.46	0.648
Female, n (%)	3 (21.4%)	9 (23.0%)	0.02	0.899
Education, y, mean ± SD	12.64 ± 2.37	12.80 ± 2.88	-0.17	0.860
Hypertension, n (%)	7 (50.0%)	27 (69.2%)	1.66	0.198
Hyperlipemia, n (%)	4 (28.5%)	16 (41%)	0.68	0.410
Diabetes, n (%)	5 (35.7%)	18 (46.1%)	0.46	0.499
History of smoking, n (%)	11 (78.5%)	26 (66.6%)	0.70	0.405
FSRP score, mean ± SD	9.50 ± 2.96	11.21 ± 4.73	-1.26	0.214
MoCA, mean ± SD	25.43 ± 1.40	14.82 ± 5.17	7.54	<0.001*
Lacune, n, median (interquartile range)	3 (1-4.25)	5 (3-9)	-4.75	0.012*
WMH volume, mL, median	3.11 (1.22-6.09)	6.66 (2.31-16.44)	-3.44	0.079
Basal ganglia EPVS score, median (interquartile range)	1 (1-2)	2 (2-2)	-1.55	0.001*
Centrum semiovale EPVS score, median (interquartile range)	1 (1-1)	1(0-1)	-0.87	0.100

PSCN, post-stroke cognitively normal; PSCI, post-stroke cognitive impairment; FSRP, Framingham Stroke Risk Profile; MoCA, Montreal Cognitive Assessment; WMH, white matter hyperintensities; EPVS, enlarged perivascular spaces; SD, standard deviation; *, the difference is statistically significant.
